# Role of ADGRG1/GPR56 in Tumor Progression

**DOI:** 10.3390/cells10123352

**Published:** 2021-11-29

**Authors:** Kwai-Fong Ng, Tse-Ching Chen, Martin Stacey, Hsi-Hsien Lin

**Affiliations:** 1 Department of Anatomic Pathology, Chang Gung Memorial Hospital-Linkou, Taoyuan 33305, Taiwan; nkf362@adm.cgmh.org.tw (K.-F.N.); ctc323@adm.cgmh.org.tw (T.-C.C.); 2 Faculty of Biological Sciences, School of Molecular and Cellular Biology, University of Leeds, Leeds LS2 9JT, UK; m.stacey@leeds.ac.uk; 3 Division of Rheumatology, Allergy, and Immunology, Chang Gung Memorial Hospital-Keelung, Keelung 20401, Taiwan; 4 Center for Medical and Clinical Immunology, College of Medicine, Chang Gung University, Taoyuan 33302, Taiwan; 5 Department of Microbiology and Immunology, College of Medicine, Chang Gung University, Taoyuan 33302, Taiwan

**Keywords:** adhesion GPCR, GPR56, ligand, signaling, tumorigenesis

## Abstract

Cellular communication plays a critical role in diverse aspects of tumorigenesis including tumor cell growth/death, adhesion/detachment, migration/invasion, angiogenesis, and metastasis. G protein-coupled receptors (GPCRs) which constitute the largest group of cell surface receptors are known to play fundamental roles in all these processes. When considering the importance of GPCRs in tumorigenesis, the adhesion GPCRs (aGPCRs) are unique due to their hybrid structural organization of a long extracellular cell-adhesive domain and a seven-transmembrane signaling domain. Indeed, aGPCRs have been increasingly shown to be associated with tumor development by participating in tumor cell interaction and signaling. ADGRG1/GPR56, a representative tumor-associated aGPCR, is recognized as a potential biomarker/prognostic factor of specific cancer types with both tumor-suppressive and tumor-promoting functions. We summarize herein the latest findings of the role of ADGRG1/GPR56 in tumor progression.

## 1. Introduction

G protein-coupled receptors (GPCRs) play a central role in cellular communication [1,2,3]. Aberrant expression and/or genetic mutations of GPCRs are often identified in various cancer types, manifesting either tumor-promoting or tumor-suppressive functions [4,5,6]. Furthermore, GPCR transactivation of different signaling molecules such as the receptor tyrosine kinases (RTKs) are closely linked to tumorigenesis [7,8]. Therefore, GPCRs and their cognate ligands as well as the specific signaling networks induced by the GPCR-ligand interaction are the key areas of interest for cancer research.

**Figure 1:**
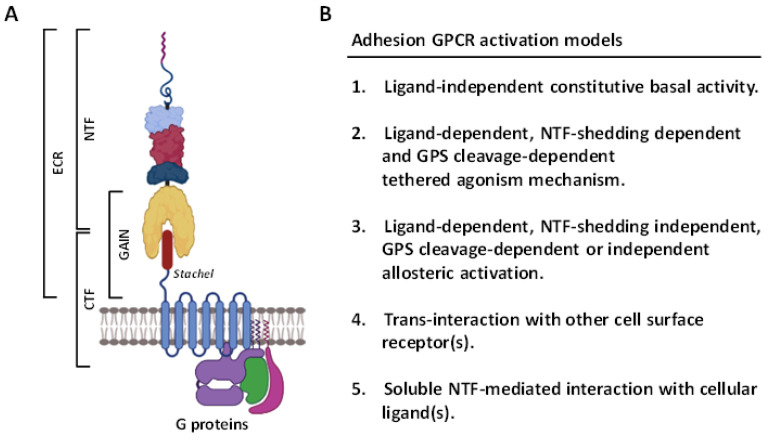
Structural features and activation mechanisms of adhesion GPCRs. (**A**) Schematic depicting the structural organization of aGPCRs in general. The ECR contains multiple cell-adhesive protein domains (indicated by different colored lines and shapes) followed by a GAIN domain. The *Stachel* peptide is depicted as a brown cylinder. ECR, extracellular region; CTF, C-terminal fragment; NTF, N-terminal fragment; GAIN, GPCR autoproteolysis-inducing. Illustration was generated using the Biorender software. (**B**) The diverse potential activation mechanisms mediated by aGPCRs.

In recent years, the GPCR superfamily has been further classified based upon their phylogenetic characteristics and subdivided into five main families, including glutamate, rhodopsin, adhesion, frizzled/Taste2, and secretin (GRAFS) [9]. Among these, the adhesion GPCRs (aGPCRs) have several unusual structural and functional features [10] (Figure 1). Firstly, the extracellular region (ECR) of most aGPCRs is uncommonly large and often consists of diverse cell-adhesive protein modules such as the epidermal growth factor (EGF)-like, immunoglobulin (Ig)-like, and lectin-like domains at its N-terminal half. These protein modules normally function as the binding sites for specific cellular ligands and/or binding partners of aGPCRs [10,11]. Secondly, the majority of aGPCRs comprise a signature GPCR autoproteolysis-inducing (GAIN) domain at the C-terminal half of ECR. A self-catalytic proteolytic reaction normally occurs during receptor biosynthesis at the consensus GPCR proteolysis site (GPS) within the GAIN domain [12,13,14]. As a result, GPS cleavage disjoints the receptor into a non-covalent dual-subunit complex comprising an N-terminal extracellular fragment (NTF) and a C-terminal 7TM fragment (CTF) [12,13,14] (Figure 1A). Thirdly, the activation and signaling mechanisms of aGPCRs are multifaceted, including the GPS cleavage-dependent and -independent as well as NTF-CTF dissociation-dependent and -independent modes [10,15,16,17]. Nevertheless, a tethered agonism model is well accepted as the common activation mechanism for most aGPCRs [18,19]. This involves the unmasking and binding of a tethered agonistic *Stachel* peptide with its own 7TM, following the separation of NTF from CTF caused by the binding of its cellular ligand(s) to the ECR [20] (Figure 1B).

Due to the structural complexity and functional diversity, the study of aGPCRs is still in its infancy. The human aGPCR family consists of 33 distinct members, some of which have been increasingly linked to various aspects of cancer biology [21,22,23]. In this review article, we summarize the molecular and functional properties of ADGRG1/GPR56 and discuss its diverse roles in tumor progression.

## 2. Overview of the ADGRG1/GPR56 Receptor

### 2.1. Structural Characteristics of the ADGRG1/GPR56 Protein

The full-length human ADGRG1/GPR56 receptor is a protein of 693 amino acids encoded by the *ADGRG1* gene on the chromosome 16q21 [24]. However, extensive RNA splicing of its transcripts is predicted to generate at least five different GPR56 protein isoforms that are different in the composition of ECR and/or the first intracellular loop. These GPR56 receptor variants have been found to have differential functional and signaling characteristics and are discussed later [25,26]. Moreover, the usage of multiple alternative transcription start sites has been identified to generate many more diverse GPR56 transcript variants; however, the frequencies of these transcripts and the resulting protein isoforms remain to be fully investigated [27,28].

**Figure 2:**
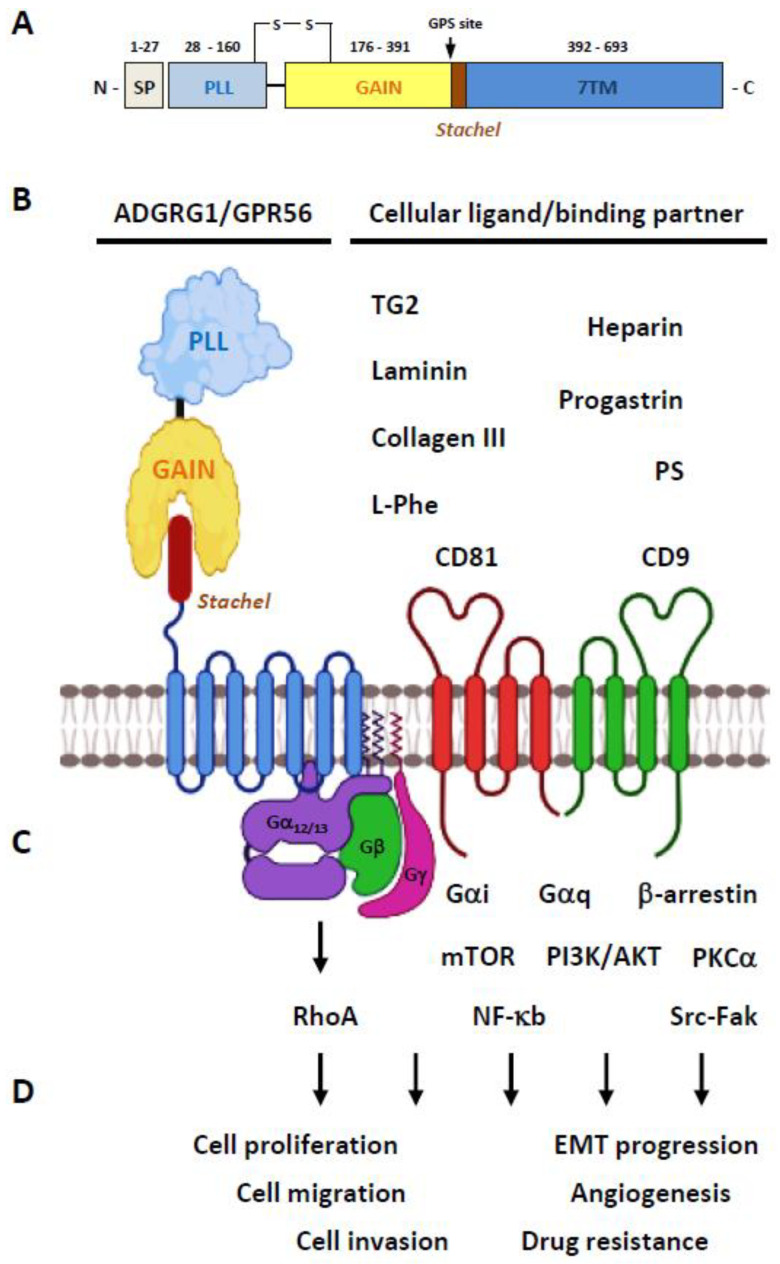
Overview of the ADGRG1/GPR56 protein. (**A**) Schematic depicting the domain organization of the GPR56 receptor protein. The light-purple and light-yellow rectangles represent the PLL and GAIN domains, respectively. The 7TM region is represented by a light blue rectangle. The *Stachel* tethered peptide is indicated by a brown rectangle. SP, signal peptide; GPS, GPCR proteolysis site. (**B**) Diagrams showing the GPR56 receptor and its known cellular ligands and binding partners. (**C**) The GPR56-mediated signaling pathways reported to date. (**D**) The tumorigenic functions known to be mediated by GPR56. Illustration was generated using the Biorender software.

The GPR56 protein consists of a N-terminal PLL (pentraxin/laminin/neurexin sex-hormone-binding globulin-like) domain and a C-terminal GAIN domain in its ECR (Figure 2A) [26]. Of interest, the PLL domain shares only a relatively weak homology with either the pentraxin or laminin/neurexin/sex-hormone-binding globulin domain hence is a rather novel structural module unique to GPR56 [26]. The GPR56-GAIN domain is unusually small in comparison to those of other aGPCRs. Indeed, its GAIN sub-domain A contains only three α-helices compared to the six α-helices identified in the canonical GAIN sub-domain A of ADGRL1/Latrophilin-1 and ADGRB3/BAI-3 [13,26,29]. Nevertheless, the GAIN sub-domain B of GPR56 seems to be comparable with those of other aGPCRs by having 13 β-strands and 2 small α-helices. The proteolytic cleavage of GPR56 takes place between Leu^382^ and Thr^383^ at the consensus GPS motif located between the 12th and 13th β-strands of the GAIN sub-domain B [26]. Interestingly, the PLL domain is absent in two alternatively-spliced GPR56 isoforms, while the other three GPR56 variants contain essentially the same full-length ECR [25]. Altogether, GPR56 is a fully-processed archetypal aGPCR consisting of non-covalently associated NTF-CTF receptor subunits (Figure 2B).

### 2.2. Ligands/Binding Partners of the ADGRG1/GPR56 Protein

In terms of specific cellular ligands/binding partners, GPR56 was one of the first aGPCRs to be deorphanized (Figure 2B). Little et al. showed that the CD9 and CD81 tetraspanins form a complex specifically with GPR56 and many G protein subunits, including Gα_q_, Gα_11_, and Gβ [30]. This GPR56-tetraspanin-G protein complex was important for subsequent signaling regulation, hence suggesting a regulatory role for CD9 and CD81 as the membrane scaffolding proteins of GPR56. Xu and colleagues identified tissue transglutaminase (also named TG2) as the specific extracellular matrix (ECM) ligand of melanoma cells [31]. Critically, it was found that the TG2-GPR56 interaction inhibited the growth and metastasis of melanoma tumors [32,33,34]. Piao’s group subsequently discovered another ECM ligand of GPR56, the type III collagen (collagen-III), in the mouse brain [35]. The interaction of collagen-III and GPR56 activated the Gα_12/13_-RhoA signaling pathway and played an essential role in the proper lamination of the cerebral cortex in the developing brain. Later studies by the same group also identified a different ECM ligand, laminin, for GPR56 [36]. It was revealed that the GPR56 receptor in oligodendrocyte precursor cells (OPCs) responded to the microglia-derived TG2 in the presence of laminin. Recently, the PLL domain was identified as the specific binding site for collagen-III and TG2 on GPR56 [37,38].

As well as protein ligands, heparin was recently identified by Chiang et al. as a novel glycosaminoglycan (GAG) binding partner of GPR56 [39]. Interestingly, heparin-GPR56 interaction reduced the shedding of its NTF and enhanced cell adhesion and motility [39]. Jin et al. demonstrated that progastrin, an 80 aa-long precursor of the peptide hormone gastrin, bound directly to GPR56 expressed in colonic stem/progenitor cells and colon cancer cells to promote cellular proliferation [40]. More recently, Chen and colleagues screened a large number of bioactive metabolites from intestinal microbiota and identified the essential amino acid L-Phe, found abundantly in the culture supernatants of the species *B. theta* (*B. theta* C34), as a small molecule agonist of GPR56 and ADGRG3/GPR97 [41]. Functional analyses showed that the ECR of GPR56 was required for the receptor activation induced by L-Phe. Nevertheless, GPR56 activation by L-Phe required very high concentrations of L-Phe (>1 mM); hence, the physiological significance of this ligand-receptor interaction remained unanswered. Lastly, Li et al. identified phosphatidylserine (PS) as an isoform-specific ligand of the GPR56-S4 variant restrictedly expressed in microglial cells [42]. The GPR56-S4 isoform lacked the entire PLL domain, but the specific PS-S4 isoform interaction was shown to be critical for microglia-mediated synaptic pruning during brain development [42].

Finally, receptor-specific monoclonal antibodies (mAbs), polyclonal antibodies (pAbs), and monobodies have been generated to serve as the artificial ligands of GPR56 for various functional studies [43,44,45,46,47]. Moreover, 3-α-acetoxydihydrodeoxygedunin (3-α-DOG) and dihydromunduletone (DHM) were identified as a selective small chemical agonist and antagonist of GPR56, respectively [48,49,50].

### 2.3. Activation and Signaling Mechanisms of ADGRG1/GPR56

Diverse activation mechanisms of the GPR56 receptor, including the GAIN domain-dependent/-independent and GPS cleavage-dependent/-independent modes, have been identified (Figure 2C) [18,47,51,52]. However, increasing evidence suggests an unusual signaling transduction mechanism in which the GPR56-NTF acts as a repressor of the basal GPR56 signaling. Indeed, NTF-truncated GPR56 receptors are constitutively active displaying increased SRF and NFAT activities, TGF-α shedding, β-arrestin binding, and receptor ubiquitination when expressed in transfected cells [51,52]. Likewise, deletion of the PLL domain increases the signaling activity of GPR56 [26]. These results strongly suggest that the activation of GPR56 (and likely most other aGPCRs) is mostly mediated via the dissociation/conformational change of the NTF. This permits a newly exposed tethered-agonist peptide within the CTF known as the *Stachel* sequence to self-activate receptor signaling [18,19]. 

As such, in comparison to the wild-type (WT) GPR56-CTF, CTF variants of GPR56 with truncated N-terminal regions showed reduced abilities to activate the Gα_13_ protein and the serum response element (SRE)-luciferase activity in transfected cells. On the other hand, synthetic peptides of the β-strand 13 of GPR56 GAIN sub-domain B were sufficient to induce the signaling activity of GPR56 [18]. Therefore, it was established that following the dissociation of NTF, the *Stachel* peptide of GPR56-CTF was exposed to interact with its own 7TM moiety, leading to conformational changes and activation of the receptor. Most recent studies have further substantiated this novel activation mechanism by demonstrating that binding of cognate ligands such as collagen-III and activating mAbs elicit GPR56 activation and Gα_12/13_-RhoA signaling after the ligand-induced NTF-CTF dissociation [44,48,53].

Recently, it was shown that the GPR56 receptor still remains constitutively active after the removal of its entire ECR. Nevertheless, this ECR-less GPR56 receptor variant only activated NFAT and induced TGF-α shedding, but not the SRF activity. This finding thus suggested a GAIN domain-independent mechanism of GPR56 activation [51,54]. On the other hand, 3-α-DOG was able to activate the GPS cleavage-deficient GPR56-F385A mutant [48,50]. Likewise, several GPR56-specific monobodies were found to activate the same GPR56-F385A mutant efficiently [47]. These results indicated that these specific agonists bind to the ECR of GPR56 and cause a unique conformational change that activates the receptor without the need of its proteolytic modification, hence supporting the GPS cleavage-independent activation mechanism of GPR56 [55].

Similar to the various GPR56 activation mechanisms described above, diverse GPR56-mediated signaling pathways were reported. Notably, most of the GPR56 signaling studies have shown the specific involvement of the Gα_12/13_-RhoA signaling axis as mentioned previously [56]. Nevertheless, additional signaling pathways involving Gα_i_, Gα_q_, Gβγ, β-arrestin, mTOR, PKCα, NF-κb, and Src-Fak have also been described elsewhere (Figure 2C) [30,32,52,57,58,59]. In conclusion, multiple activation and signaling pathways of GPR56 have been identified that seem to depend mainly on the specific ligands and cell types studied.

### 2.4. The Biological Functions of GPR56

Due to its broad mRNA expression patterns in the brain, kidney, testis, thyroid, pancreas, skeletal muscle and the hematopoietic system (BioGPS.org), GPR56 is a functionally versatile aGPCR involved in many physiological processes. In brief, the highest GPR56 expression level was detected in CD56^+^ NK cells, although cytotoxic CD8^+^, CD4^+^, and γδ T lymphocytes also expressed significant levels of GPR56 [60,61]. In the nervous systems, GPR56 expression was detected in Cajal–Retzius cells, radial glial cells, Tuj1^+^ migrating neurons, OPCs, Schwann cells (SCs), and microglial cells [35,36,62,63,64,65]. GPR56 was identified as the most abundantly expressed GPCR in the pancreatic islets, especially β-cells [66]. GPR56 was expressed in mouse Sertoli cells as well as Mullerian duct epithelium of avian female gonads [67,68]. For the general review of the role of GPR56 in health and disease, the readers are referred to a recent review article [69].

As the single disease gene responsible for bilateral frontoparietal polymicrogyria (BFPP), GPR56 is undoubtedly best known for its essential role in normal cerebral cortical development [24,35]. Subsequent studies showed that GPR56 is also critically involved in the neuronal myelination of the CNS and peripheral nervous system (PNS), myelin repair in CNS neurons, the proliferation of OPCs, proper radial axonal sorting by SCs, microglia-mediated synaptic pruning, and antidepressant response [36,62,63,64,70,71].

In addition to the nervous systems, GPR56 has been shown to play a regulatory role in myoblast fusion and mechanical overload-induced muscle hypertrophy, NK cell cytotoxic activity, hemostatic shear-force sensing by platelets, pancreatic islet function, the development and differentiation of hematopoietic stem/progenitor cell, male gonad development in mice, as well as the normal development of avian embryonic Müllerian duct [43,59,66,67,68,72,73,74,75,76,77,78,79]. 

Importantly, GPR56 was often detected and implicated in the development of many different types of cancers, including melanoma, breast, non-small cell lung, esophageal squamous cell cancer [80], ovarian, colon, pancreatic carcinoma [81,82], glioblastoma/astrocytoma [83], and leukemia [79,84,85]. In general, GPR56 was shown to regulate cell growth, adhesion, and migration of diverse cancer cell types. In addition, a role for GPR56 in modulating the epithelial-mesenchymal transition (EMT), angiogenesis, and chemo-/radioresistance of cancer cells has been reported (Figure 2D). Overall, increasing evidence has indicated that GPR56 might function as the biomarker/prognostic factor of certain cancers and a potential tumor-promoter or tumor-suppressor for others.

## 3. ADGRG1/GPR56 as a Cancer Marker and/or Prognostic Factor

Due to its restricted expression in certain stages of tumor development or the association of its expression levels with the metastatic stage or survival rate of cancer patients, GPR56 was thought to act as a potential biomarker and/or prognostic factor of certain cancers. Identified independently by Liu et al. and Zendman et al. in 1999, GPR56 (also named TM7XN1) transcripts were found to be expressed differentially in human melanoma cell lines [86,87]. Notably, GPR56 was strongly expressed in less metastatic melanoma cells, while markedly reduced in the highly metastatic ones. Therefore, the expression levels of GPR56 transcripts seemed to correlate inversely to the metastatic potentials of melanoma cells. Subsequent studies indeed showed a similar contrary relationship between GPR56 protein expression and tumor metastasis of the melanoma lesions [31,32,33]. Nevertheless, more studies are needed to fully verify the potential role of GPR56 as a negative metastatic marker of human melanoma cells.

Saito et al. found that GPR56 expression was positively regulated by the ecotropic viral integration site-1 (EVI1) transcription factor in acute myeloid leukemia (AML) cells. As such, GPR56 was highly expressed in EVI1^high^ AML cells that displayed strong cell adhesion and antiapoptotic activities [79]. GPR56 gene silencing in AML cells resulted in reduced cell growth and increased apoptosis. In addition, Gpr56 was shown to be involved in the normal development and repopulating ability of HSCs in mice. As EVI1 was implicated in regulating the stemness of leukemia cells, GPR56 was suggested as a potential therapeutic target for EVI1^high^ AML [79].

Consistent with this, Pabst et al. later identified GPR56 as a reliable leukemia stem cell (LSC) biomarker for most primary human AMLs by using next-generation sequencing and in vivo analyses of LSC frequencies. Moreover, the GPR56 expression level in LSCs was positively correlated with high-risk AML groups and poor clinical outcomes [85]. Daga et al. subsequently showed that GPR56 is the prominent surface marker of the LSC-enriched CD34^+^CD38^−^ AML cells, while Daria et al. confirmed the association of high GPR56 expression with the inferior prognosis of AMLs [88]. Experiments involving the adoptive transfer of GPR56-expressing cells significantly promote leukemia development and reduce survival rate in mice. In addition, the inhibition of AML cell engraftment by GPR56-specific Abs further supports a causal link between GPR56 and cancer progression [79,84,85]. In conclusion, GPR56 not only is a valid LSC marker and a disadvantageous prognostic factor of AML but also a potential anti-leukemic therapeutic target.

Liu et al. showed that GPR56 is an independent unfavorable prognostic factor of epithelial ovarian cancer (EOC) by investigating its immunohistochemistry expression in 110 ovarian serous carcinoma samples. They found that the GPR56 expression level was significantly associated with the advanced FIGO (International Federation of Gynecology and Obstetrics) stage and positive lymph node invasion of EOC [82]. Likewise, Lim et al. examined GPR56 expression levels in the tissue samples of colorectal cancer (CRC) patients by immunohistochemistry and found that the GPR56^high^ expression group had a lower 5-year overall survival rate than the GPR56^low^ expression group, suggesting that the GPR56 expression level might be a prognostic indicator of CRC [89]. Zhang et al. further demonstrated that GPR56 is involved in modulating the plasticity of cancer stem cells (CSC) of CRC to a more drug-resistant phenotype. It was believed that GPR56 promoted drug resistance of CRC by upregulating multidrug resistance protein 1 (MDR1) expression via a RhoA-dependent signaling pathway [90] (Table 1).

While the role of GPR56 as unique marker and/or prognostic factor of certain cancer types was strongly implicated, several caveats need to be considered. For example, a systematic evaluation of GPR56 protein expression in human cancer cells and tissues is yet to be conducted. As the splice variants and protein isoforms of GPR56 are not examined in most cancer studies, the possible impact of these receptor variants in the progression of different tumors remains obscure. Thus, the exact GPR56 RNA transcript and protein variants in different stages of tumor development need to be investigated fully. The results of these studies shall provide better insights into the understanding of the role of GPR56 as unique marker/prognostic factor of certain malignancy.

## 4. The Tumor-Suppressive Role of ADGRG1/GPR56

As mentioned above, the expression levels of GPR56 transcripts were found to correlate inversely to the metastatic potentials of human melanoma cell lines, thus suggesting a possible suppressive role for GPR56 in melanoma metastasis. The following studies, mainly conducted by Xu’s group, have shown that the interaction of GPR56 and its ECM ligand, TG2, inhibited the growth, angiogenesis, and metastasis of melanoma cells in vivo [31,32,33]. Specifically, the secretion of vascular endothelial growth factor (VEGF) was inhibited as a result of GPR56-TG2 interaction, thereby reducing the blood vessel formation at the tumor xenografts [32]. It was further shown that a serine threonine proline-rich (STP) segment of the GPR56-NTF mediated its interaction with TG2 specifically and deletion of the STP segment lead to GPR56 activation via a protein kinase C (PKC) α-dependent pathway to promote VEGF production and tumor angiogenesis [32].

The authors subsequently further uncovered a novel antagonistic relationship between GPR56 and TG2 during melanoma progression. Thus, the ECM cross-linking enzyme TG2 was shown to promote melanoma growth due to its enzyme activity, but this tumor-promoting effect was antagonized by GPR56-mediated internalization and degradation of TG2 [33]. The GPR56-modulated TG2 degradation resulted in the reduced deposition of fibronectin, a major ECM protein, and focal adhesion kinase (FAK), leading to changed ECM compositions and cell-ECM adhesion in tumor tissue microenvironments [33]. It was believed that the modified ECM in part obstructed melanoma metastases and expansion. Altogether, these results point to an inhibitory role for GPR56 in the progression of melanoma lesions. Contrarily, our recent data on the cellular functions of GPR56 in human melanoma cell lines has shown that activation of GPR56 receptor by an immobilized agonistic mAb promoted cell migration and invasion via the production of inflammatory cytokine, IL-6 [44]. These discrepancies may be due to the use of different melanoma cell lines and experimental set-ups, distinct fates of the receptor subunits and differential receptor signaling activities induced by different ligands/binding partners. Therefore, the tumorigenic role of GPR56 during melanoma development requires further investigation.

Shashidhar et al. discovered that GPR56 was highly expressed in glioblastoma multiforme/astrocytoma tumors [83]. In addition, co-localization of GPR56 and α-actinin was detected on the leading edges of the glioblastoma cell membranes. Cellular adhesion was inhibited upon cultured with immobilized recombinant GPR56-ECR, resulting in unusual cytoskeletal organization and cell rounding, suggesting a role for GPR56 in cellular adhesion and migration. Ohta et al. developed agonistic Abs specific to GPR56-ECR and showed that GPR56 activation in the U87-MG human glioma cell line by these agonistic Abs lead to retarded cell migration via a Gα_q_-Rho signaling pathway [45].

More recently, Moreno et al. reported that GPR56 played a restrictive role in the mesenchymal differentiation and radioresistance of glioblastoma (GBM) cells [57]. Based on the gene expression and epigenetic profiles, a total of five different subtypes of adult GBM were classified including the glioma-CpG island methylator phenotype (G-CIMP), proneural, neural, classical, and mesenchymal [91,92,93]. In general, the poorest prognosis was found in patients of the non G-CIMP GBM subtypes, while the G-CIMP GBM patients usually showed a more favorable prognosis [91]. It was well known that the non-G-CIMP GBMs can transform intrinsically from one subtype to another, and the mesenchymal subtype was correlated more strongly with higher radioresistance and shorter survival prognosis [94,95]. Of importance, a mesenchymal transition of GBMs also occurred in response to therapy.

GPR56 was found to be strongly expressed in proneural and classical GBM cells, but its expression was greatly reduced, even lost, during the transition of these cells toward a mesenchymal phenotype. Moreover, in vitro and in vivo mesenchymal differentiation and radioresistance were enhanced in GPR56-knockdown glioma-initiating cells [57]. As a result, a low GPR56-associated transcriptomic signature was associated with a poor outcome in GBM patients. It was shown that GPR56 might impede mesenchymal differentiation and radioresistance in part via the inhibition of the NF-κB signaling pathway in GBM cells. Finally, a positive correlation was identified between a low GPR56-associated signature and the mesenchymal phenotype-related signatures and inflammatory signatures of multiple tumor types in addition to GBMs. Conversely, a negative correlation was noted between a low GPR56-associated signature and those related to epithelial differentiation and cell proliferation. Hence, GPR56 is likely to function as a potential tumor-suppressor by inhibiting the epithelial-mesenchymal transition (EMT) in certain cancer cells [57] (Table 2).

Taken together, GPR56 likely exerts tumor-suppressive functions in a tumor cell-type specific manner via several signaling pathways including PKCα, Gα_q_-Rho, and NF-κB. Interaction with TG2 seems to be a critical GPR56 activation mechanism in melanoma cells, but its interacting ligand(s) in glioblastoma cells remains uncharacterized. Interestingly, both melanoma and glioblastoma cells are mostly derived from the neuroepithelial tissues originated from multipotent neural crest cells. The relationship between the tumor-suppressive role of GPR56 and the common embryonic origin of tumor cells is an interesting area of future research.

## 5. The Tumor-Promoting Role of ADGRG1/GPR56

Contrary to the tumor-suppressive functions discussed above, a potential tumor-promoting/oncogenic function has been reported for GPR56 in diverse cancer types. Therefore, in comparison to the surrounding normal tissues, upregulated GPR56 expression is detected in many human cancer tissues, including breast and pancreatic cancers, colorectal, renal, cervical, esophageal squamous cell carcinoma (ESCC), non-small-cell lung carcinoma (NSCLC), and epithelial ovarian tumors [80,81,82,90,96,97]. In general, upregulated GPR56 expression was associated with enhanced cell growth, adhesion, migration, and/or drug resistance of cancer cells.

Ke et al. reported a positive correlation between higher GPR56 expression levels and the transformation phenotypes of several cancer cell lines [81]. As such, GPR56-specific gene silencing leads to enhanced apoptosis and reduced anchorage-independent growth of cancer cells. By contrast, GPR56 overexpression in mouse fibroblast NIH3T3 cells promoted cellular transformation and increased focus formation due to the loss of contact inhibition. Finally, the tumorigenic role of GPR56 was analyzed in several in vivo models of tumor xenograft using melanoma, colon, and prostate cancer cell lines. By inducing GPR56 gene silencing at different stages of the in vivo tumor xenograft models and examining the final tumor burdens, it was concluded that GPR56 plays a significant role in promoting tumor growth at the early as well as advanced stages of tumor progression.

Similarly, forced GPR56 expression was found to promote the proliferation, migration, and/or invasion of CRC, EOC, osteosarcoma, NSCLC, and prostate cancer cells by several groups [80,82,90,96,98,99]. In contrast, GPR56 gene silencing resulted in increased cell apoptosis and suppression of cell proliferation, migration, invasion, and/or tumor growth of these cancer cells. Furthermore, Ji et al. demonstrated that GPR56 might enhance the metastasis of CRC cells by promoting the EMT process via the induction of PI3K/AKT signaling pathway [96]. Interestingly, concomitant expression of GPR56, TG2, and NF-κB was detected in ESCCs, and a significant correlation was found between their expression levels and the nodal invasion and metastasis of ESCCs [80,97]. As mentioned earlier, our recent study in human melanoma cells showed that GPR56 activation induced IL-6 secretion, promoting cell migration and invasion via the Gα_12/13_-RhoA pathway [44]. In a latest publication, Sasaki et al. showed that GPR56 played a crucial role in promoting bone metastasis of breast cancer cells in a GPS proteolysis-dependent fashion by interacting with collagen-III in metastasis sites [100].

The CSC subpopulation of CRC was marked specifically by the expression of Leucine-rich repeat-containing G-protein coupled receptor 5 (LGR5) and LGR5^+^ CSCs cells were able to transform to a more drug-resistant LGR5^−^ phenotype. Zhang et al. identified significantly increased GPR56 expression in drug-resistant LGR5^−^ CRC cells compared to LGR5^+^ CSCs. The GPR56^+^LGR5^-^ CRC cells were more resistant to irinotecan and 5-fluorouracil, likely due to the upregulated MDR1 expression induced by GPR56 via a RhoA-dependent signaling mechanism [90]. Consistently, GPR56 knockdown reduced MDR1 expression in CRCs leading to increased sensitivity to chemotherapy and impeded tumor growth. In contrast, forced GPR56 overexpression in CRCs resulted in increased tumor growth in vivo. Finally, the expression levels of GPR56 transcript were much higher in primary colon tumors than in matched normal tissues and were correlated with poor survival outcomes. Hence, GPR56 seems to be associated with the CSC plasticity and drug resistance of colorectal tumors [90]. 

LSCs are the rare leukemia-initiating cells and represent the main driver for disease relapse. As mentioned, GPR56 was identified as a stable LSC marker for the majority of AML samples and that its expression was regulated in part by EVI1 in certain AML cells [79,85]. Notably, EVI1-regulated GPR56 was associated with the enhanced cell adhesion and antiapoptotic phenotypes observed in EVI1^high^ human AML cells. Conversely, GPR56 knockdown in AML cells resulted in increased cell migration and decreased cell adhesion to a wide variety of ECM proteins via a RhoA-dependent signaling pathway. In addition, strong Gpr56 expression was detected in murine HSCs and its expression was gradually reduced during hematopoietic differentiation. Interestingly, the number of HSCs was greatly reduced in the bone marrow (BM), but increased in the spleen, liver, and peripheral blood of Gpr56-deficient mice, suggesting a role for Gpr56 in the maintenance of HSCs in BM. Indeed, the in vivo repopulating ability of the Gpr56^−/−^ HSCs was significantly impeded, likely due to their decreased cell adhesion and increased cell migration abilities. Hence, Gpr56 might be involved in the BM retention and peripheral trafficking of HSCs [79].

GPR56 expression in LSCs was higher than that in HSCs. Furthermore, a positive correlation was established between a higher GPR56 expression level and the LSC gene signature, as well as the high-risk AML patients and poor clinical outcomes [84,85]. Likewise, significantly high GPR56 expression was identified in AML cells of patients with mutant nucleophosmin 1 (NPM1) and FMS-like tyrosine kinase 3 (FLT3)-length mutation. Jentzsch et al. recently further showed a positive link between the high GPR56 expression at diagnosis and a higher relapse risk of AML patients receiving allogeneic HSC transplantation [101]. In summary, GPR56 expression seems to be closely associated with the disease progression of myeloid leukemia. 

The functional role of GPR56 in myeloid leukemogenesis was validated using murine hematopoietic progenitor cells (HPCs) co-expressing homeobox A9 (HOXA9) and Gpr56, which increased primary colony formation in vitro and accelerated myeloid leukemogenesis in vivo compared to HPCs expressing HOXA9 alone [84]. Conversely, the development of leukemia in vivo was greatly delayed in mice transplanted with HOXA9/Meis1-transduced cells in which the Gpr56 expression was knock-downed by gene-specific shRNAs. Importantly, leukemic engraftment of the NOD scid gamma (NSG) mice by the HOXA9-expressing MV4-11 AML cell line was impaired significantly following the functional blockage of GPR56 by an anti-GPR56 mAb. Taken together, these findings demonstrate clearly that GPR56 contributes positively to AML development and identify GPR56 as a potential Ab-based therapeutic target in AML (Table 3).

In summary, GPR56 is upregulated in many cancer types and functions as a potential tumor-promoting receptor, modulating cell growth, apoptosis, migration/invasion, EMT process, and/or drug resistance. The upregulated GPR56 expression in cancer is cell type-specific and is regulated in part by unique transcription factors such as EVI1. Several signaling pathways such as the PI3K/AKT and Gα_12/13_-RhoA axes are implicated in its tumor-promoting functions; however, the cellular ligand(s) and the exact receptor activation mechanisms involved require further studies. In contrast to the neural crest origin of melanoma and glioblastoma, the origins of these carcinoma and LSC/HPC cells are of epithelial and hematopoietic lineages, respectively. It is hence possible that the tumor-promoting or tumor-suppressive functions of GPR56 is determined in part by the differential signaling responses of tumor cells of different origins.

## 6. Unmet Challenges

While the roles of GPR56 in tumor development have been increasingly recognized, more detailed investigation is required in several areas of research. As elucidated previously, earlier studies have not addressed the possible involvement of diverse GPR56 transcripts and receptor isoforms nor the signaling pathways in tumor progression. Considering the extensive GPR56 RNA splicing and exon usage, many potential GPR56 variants with different ligand-binding and signaling functions could be expressed in tumor cells. Hence, it will be of importance to know if some unique receptor RNA transcripts and isoforms such as the smaller 1st intracellular loop-containing GPR56-S1 and the PLL-truncated GPR56-S4 variants are expressed in more abundance in certain cancer types. The use of single-cell RNA sequencing analysis perhaps will help answer this question and pave the way for the understanding of whether alternative splicing of GPR56 modulate its tumorigenic functions. These studies might be extended to explore whether certain splice variants could predict and therefore be used to diagnose tumor outcomes better than the full-length GPR56. In view of the close association of disease-causing GPR56 mutations with BFPP, the impact of GPR56 mutations in tumorigenesis will be an important direction for future investigation. 

Similarly, the role of GPR56-specific cellular ligands in different tumors has not been delineated in detail. The results of several studies have shown that GPR56-TG2 interaction impeded melanoma growth, angiogenesis and metastasis, whereas GPR56 binding to collagen-III promoted the metastasis of breast cancer cells to bone [31,32,33,100]. Thus, the interaction of GPR56 and its specific ligands clearly plays a critical role in the functional modulation of tumor cells and therefore needs deeper investigation. Most current studies relied heavily on the levels of GPR56 transcripts in solid tumor samples with minimum data of GPR56 protein expression. Moreover, the overall evaluation of the quality of protein expression studies in human cancer samples is not applicable because of the use of different reagents such as monoclonal and polyclonal Abs by different researchers. Indeed, anti-GPR56 Abs used in various solid tumor studies were polyclonal Abs developed in different animal species such as rabbit, mouse, goat, and sheep [82,83,89,98]. The Ag fragments used for Ab generation included different lengths of GPR56-ECR peptides as well as the full-length GPR56 protein expressed in transfected cells. In addition, the quantitative analyses of GPR56 expression in solid tumors as revealed by immunohistochemical analysis required careful evaluation of the different stages of cancer samples, the experimental procedures of tissue section preparation, as well as Ag retrieval and Ab staining conditions. It is hence difficult to compare GPR56 protein expression data in a systematic and comprehensive fashion among different published results. For example, in the study of the potential tumor-suppressor role of GPR56 in GBM, goat-derived GPR56 polyclonal Abs were used mainly in western blotting analyses with only one immunohistochemical staining result [57]. By contrast, mouse anti-GPR56 polyclonal Abs were employed exclusively in western blotting analyses of CRC cell lines to examine its possible tumor-promoting role [90]. No immunohistochemical analysis of GPR56 expression was investigated in this study. It is therefore essential to develop robust Ab reagents in the future for the detection of GPR56 receptor isoforms in situ.

GPS auto-proteolysis is a unique post-translational modification important for certain functional aspects of aGPCRs. As discussed, GPS cleavage-dependent and -independent functions have been described for GPR56. Nevertheless, the involvement of GPS auto-proteolysis in the tumorigenic role of GPR56 has not yet been studied fully. In addition, the possible role of GPR56 *cis*-interacting proteins such as CD9 and CD81 in modulating the tumorigenic functions of GPR56 also requires further attention. Finally, there are very few studies focusing on the regulation of GPR56 expression by specific transcription factors in cancers. Answers to these questions will not only delineate the regulatory mechanisms of GPR56′s functions in tumorigenesis but also help reveal the paradoxical tumor-promoting and tumor-suppressive roles of GPR56 in different cancer types.

## 7. Conclusions and Future Perspective

ADGRG1/GPR56 is undoubtedly an important aGPCR relevant in tumor development. However, its role in tumorigenesis seems to depend critically on tumor cell type- and/or stage-specific contexts. This conclusion is not only backed up by the divergent results from multiple human cancer types discussed above, but also from animal tumor models of endogenous cancer progression [102,103]. As such, the development of GPR56-specific agonists and antagonists will be needed for the deeper understanding of its functional role in different cancer types. In addition, the development of specific reagents such as the pyrrole-imidazole polyamides that disrupted the binding of EVI-1 to the GPR56 promoter is an alternative therapeutic approach to modulate GPR56 expression in certain cancer types [104]. Future studies also need to dissect further the exact signaling pathways and the ligands/interacting partners of GPR56 at specific stages of tumor development in order to identify the molecular targets of which therapeutic intervention can be devised. Nevertheless, with the realization of it being a specific surface marker of unique cancer cell types such as the LSC of AML, GPR56 may be an ideal molecular target for the combinatorial chimeric antigen receptor (CAR) therapy against AML [105].

## Figures and Tables

**Table 1:** ADGRG1/GPR56 as a cancer marker and/or prognostic factor.

Cancer Type	Function	References
Melanoma	Potential negative metastatic marker/factor	[31,32,33,86]
Acute myeloid leukemia	Leukemia stem cell marker Unfavorable prognostic factor	[79,84,85,88]
Epithelial ovarian cancer	Unfavorable prognostic indicator	[82]
Colorectal cancer	Unfavorable prognostic indicator Promote drug-resistant cancer stem cell	[89,90]

**Table 2:** The tumor-suppressive role of ADGRG1/GPR56.

Cancer Type	Potential Mechanisms	References
Melanoma	Binding of the ECM TG2 ligand inhibited tumor growth, angiogenesis, and metastasis due to GPR56-mediated TG2 internalization and degradation, reduced deposition of fibronectin in ECM, and reduced production of VEGF via a PKCα-dependent pathway.	[31,32,33]
Glioblastoma	Inhibitory effects on cell adhesion and migration via Gα_q_-Rho signaling. A restrictive role in mesenchymal differentiation and radioresistance due in part to the inhibition of the NF-κB signaling pathway.	[45,57,81]

**Table 3:** The tumor-promoting role of ADGRG1/GPR56.

Cancer Type	Potential Mechanisms	References
Breast, pancreatic, cervical, ovarian, prostate, and colorectal cancers, non-small-cell lung carcinoma (NSCLC), and esophageal squamous cell carcinoma	Upregulated GPR56 expression in cancer cells promoted cell growth, adhesion, migration, and/or drug resistance of cancer cells.	[80,81,82,90,96,97,98,99,100]
Colorectal cancer (CRC)	GPR56 promoted the EMT process via the induction of PI3K/AKT signaling pathway. GPR56 upregulated MDR1 expression via a RhoA-dependent signaling pathway.	[90,96]
Melanoma	GPR56 activation upregulated IL-6 production, promoting cell migration	[44]
Acute myeloid leukemia (AML)	GPR56 enhanced cell adhesion and antiapoptotic functions a RhoA-dependent signaling pathway. Gpr56^+^ HPCs increased primary colony formation in vitro and accelerated myeloid leukemogenesis in vivo.	[79,84,85]

## Data Availability

Not applicable.
